# Multifunctional peptides derived from an egg yolk protein hydrolysate: isolation and characterization

**DOI:** 10.1007/s00726-014-1869-x

**Published:** 2014-11-20

**Authors:** Aleksandra Zambrowicz, Marta Pokora, Bartosz Setner, Anna Dąbrowska, Marek Szołtysik, Konrad Babij, Zbigniew Szewczuk, Tadeusz Trziszka, Gert Lubec, Józefa Chrzanowska

**Affiliations:** 1 Department of Animal Products Technology and Quality Management, Faculty of Food Science, Wrocław University of Environmental and Life Sciences, Chełmońskiego 37/41, 51-630 Wrocław, Poland; 2 Faculty of Chemistry, University of Wrocław, Wrocław, Poland; 3 Department of Pediatrics, Medical University of Vienna, Währinger Gürtel 18, 1090 Vienna, Austria

**Keywords:** Egg yolk protein by-product, Hydrolysis, Antioxidant, Antidiabetic, Angiotensin I-converting enzyme inhibitory activity

## Abstract

An egg yolk protein by-product following ethanol extraction of phospholipids (YP) was hydrolyzed with pepsin to produce and identify novel peptides that revealed antioxidant, ACE inhibitory and antidiabetic (α-glucosidase and DPP-IV inhibitory) activities. The peptic hydrolysate of YP was fractionated by ion-exchange chromatography and reversed-phase high-pressure liquid chromatography. Isolated peptides were identified using mass spectrometry (MALDI-ToF) and the Mascot Search Results database. Four peptides of MW ranging from 1,210.62 to 1,677.88 Da corresponded to the fragments of Apolipoprotein B (YINQMPQKSRE; YINQMPQKSREA), Vitellogenin-2 (VTGRFAGHPAAQ) and Apovitellenin-1 (YIEAVNKVSPRAGQF). These peptides were chemically synthesized and showed antioxidant, ACE inhibitory or/and antidiabetic activities. Peptide YIEAVNKVSPRAGQF exerted the strongest ACE inhibitory activity, with IC_50_ = 9.4 µg/mL. The peptide YINQMPQKSRE showed the strongest DPPH free radical scavenging and DPP-IV inhibitory activities and its ACE inhibitory activity (IC_50_) reached 10.1 µg/mL. The peptide VTGRFAGHPAAQ revealed the highest α-glucosidase inhibitory activity (IC_50_ = 365.4 µg/mL). A novel nutraceutical effect for peptides from an egg yolk hydrolysate was shown.

## Introduction

Diet-related diseases such as cardiovascular disease, obesity, hypertension and diabetes mellitus (DM) represent significant health problems worldwide and there is evidence to suggest a possible common pathophysiology among type 2 DM, cardiovascular disease and oxidative stress.

DM is an endocrine system disease that causes metabolic disorders leading to multiple organ damage syndromes. The most effective therapy for DM is the maintenance of optimal blood glucose levels (Matsui et al. 1996; Yu et al. 2012). Accordingly, an effective strategy for the control of DM type 2 is the use of inhibitors of α-glucosidase which catalyzes the cleavage of d-glucose from oligosaccharides and disaccharides, and the inhibitors of dipeptidyl peptidase-4 (DPP-4) which participates in the rapid decomposition of GLP-1, a hormone increasing the secretion of insulin by the pancreatic beta cells and decreasing the secretion of glucagon after meal (Matsui et al. 1996; Lacroix and Li-Chan 2012). DM type 2 can lead to serious complications including organs damage, such as renal failure. Insulin resistance is also related to hypertension (Matsui et al. 1996).

Hypertension is one of the major risk factors for cardiovascular disease (Miguel et al. 2004). Substances displaying inhibitory activity against dipeptidyl carboxypeptidase (angiotensin-converting enzyme or ACE, EC 3.4.15.1) play an important role in controlling the development of hypertension by regulating the renin–angiotensin system. Angiotensin I is a substrate for ACE which produces angiotensin II by removing the C-terminal dipeptide (HL) from angiotensin I. Angiotensin II, by causing muscle contraction in small blood vessels, significantly raises the blood pressure and increases the heart rate. ACE also removes the C-terminal dipeptide of bradykinin (a vasodilator), resulting in the creation of an inactive peptide fragment (Ondetti and Cushman 1999). Therefore, inhibition of ACE activity is considered an effective approach to control hypertension.

Reactive oxygen species (ROS) are natural by-products of normal oxygen metabolism and have important roles in cell signaling and homeostasis. ROS have a significant role including inactivation of nitric oxide (Cai and Harrison 2000). However, dramatic increases in ROS result in significant damage to human cells by altering proteins, lipids and DNA. The imbalance in redox state causes oxidative stress, endothelial dysfunction and reduction of NO availability (Hadi et al. 2005). Finally, this leads to several simultaneous processes which may culminate in vascular damage and hypertension (Touyz et al. 2003).

Antioxidants, as well as peptides, are compounds capable of inhibiting oxidative reactions and protecting the human body against the destructive activity of free radicals. Numerous studies have been undertaken to develop novel bioactive peptides from food proteins. The aforementioned dependencies between the etiologies of various diseases indicate the special importance of multifunctional peptides: their combined antioxidant, ACE inhibitory and antidiabetic properties would make a useful peptide preparation for the control and prevention of diet-related diseases.

There is a need for natural and easily bio-renewable materials that could be used to obtain bioactive food additives and medicines used in the treatment of various medical conditions (Mine and Kovaks-Nolan 2006; Yu et al. 2012). One example of such material is egg yolk, providing well-known egg-derived bioactive substances such as lysozyme, cystatin, avidin, phosvitin and phospholipids. Recently, there has been a significant increase in studies of modification of egg components to improve or evaluate other biological activities. Representative examples include dimerization of lysozyme, egg phosphatidylcholine enrichment in polyunsaturated fatty acids and enzymatic hydrolysis of ovalbumin (Lesnierowski et al. 2004; Gładkowski et al. 2011; Yu et al. 2011).

Enzymatic hydrolysis is one of the most important and widely used technologies in obtaining bioactive peptides from egg proteins. These peptides, released from the proteins by the action of proteolytic enzymes, have various beneficial health effects in the circulatory, nervous and immune systems. Egg protein-derived bioactive peptides include opioid peptides, peptides lowering high blood pressure, inhibiting platelet aggregation, carriers of metal ions and peptides with immunostimulatory, antimicrobial, antidiabetic and antioxidant activity (Mine and Kovaks-Nolan 2006; Yu et al. 2011).

In recent years, researchers in this area have been looking for unconventional high-performance enzymes, developing hydrolysis methods for the isolation of peptides and technologies based mainly on the use of native proteins. In addition, the preparation of bioactive peptides by enzymatic hydrolysis of proteinaceous by-products could become an interesting method of waste disposal. It also seems desirable to be able to use the entire egg as a source of biologically active substances, including any by-products generated in the process of isolation of these substances.

Nowadays, egg yolks are intensively used for the extraction of valuable phospholipids. Delipidated egg yolk proteins (YP) are the main by-products of lecithin extraction from egg yolk. The delipidation process, using ethanol and hexane for the removal of phospholipids, impairs protein functionality and so the YP possess limited biological and biotechnological value (Jiang et al. 2001; Ting et al. 2011). YP by-products, however, can be converted into value-added products with functional and biological properties improved by enzymatic hydrolysis.

The aim of the study was to obtain peptides with antioxidant and ACE inhibitory activities as well as antidiabetic properties, via enzymatic hydrolysis of a by-product that had been generated from the isolation of egg yolk phospholipids, and to confirm their biological properties.

## Materials and methods

### Substrate

40–45-week-old Lohmann brown line laying hens were housed in a bedding system. Eggs were automatically broken open and macroscopic parts were separated on an industrial scale. An egg yolk protein preparation (YP), the by-product of phospholipid extraction using ethanol (Siepka et al. 2010), was used as a substrate. The YP was dephosphorylated before enzymatic hydrolysis (Xu et al. 2007) and was then lyophilized and stored frozen until further use.

### Enzyme

Enzymatic hydrolysis was performed using commercially available pepsin (EC. 3.4.23.1) from porcine stomach type A (Sigma P7012).

### Determination of proteolytic activity

Proteolytic activity was determined in a reaction with 2 % hemoglobin as a substrate and incubated for 10 min at 37 °C. The reaction was halted by the addition of 5 % trichloroacetic acid (TCA). The samples were then centrifuged, and the absorbance of supernatants was measured at *λ* = 280 nm. One unit of enzymatic activity (U) was defined as the amount of enzyme giving an increase in absorbance at 280 nm of 0.1 under reaction conditions.

### Determination of protein content

The total protein (*N* × 6.25) in the insoluble substrate was determined using the Kjeldahl method. Protein content in hydrolysates and peptide fractions were determined by the Lowry et al. method (1951) using BSA (Sigma P0834) as a standard.

### Enzymatic hydrolysis

YP hydrolysis was carried out according to a modified method by Zambrowicz et al. (2013). A substrate suspension (10 %) in 0.02 M Gly-HCl buffer (pH 3.5) was hydrolyzed at 37 °C for 2 h using pepsin at a dose of 20 U/mg of hydrolyzed YP. The reaction was quashed by heating at 100 °C for 10 min. The hydrolysate was cooled, centrifuged (5,500×*g*, 10 min, 10 °C) and the supernatant was lyophilized and stored at 4 °C until further use.

### The degree of hydrolysis

Degree of hydrolysis (%) was determined by measuring TCA soluble protein (Silvestre 1996).

### Determination of ACE inhibitory activity

The ACE (EC 3.4.15.1; from rabbit lung, Sigma, A6778) inhibitory activity of hydrolysate and peptide fractions was measured spectrophotometrically according to Miguel et al. (2004). A hydrolysate solution (40 µL) mixed with a Hippuryl-His-Leu (HHL) (Sigma H4884) solution (5 mM in 100 mM potassium phosphate containing 300 mM sodium chloride, pH 8.3) as substrate was preincubated at 37 °C for 5 min, and the reaction was initiated by adding 20 µL (2 mU) of ACE solution and incubating for 30 min at the same temperature. The enzymatic reaction was halted by the addition of 150 µL of 1 M HCl. The liberated hippuric acid was extracted using 1 mL of ethyl acetate and vigorous shaking, after which 750 µL of the upper layer was transferred into a test tube and evaporated in a vacuum. The hippuric acid left in the tubes was re-dissolved in 800 µL of distilled water and the absorbance was measured at *λ* = 228 nm. All samples were tested in three replications. The inhibition activity was calculated using the following equation:

Inhibition activity (%) = [(*A* − *B*)/*A*] × 100 %

where *A* is the reaction blank, of which the mixture contained the same volume of the buffer solution instead of the ACE inhibitor sample; *B* is the reaction in the presence of both ACE and its inhibitor. The IC_50_ value was estimated from a dose–response curve of an inhibitor versus the ACE activity.

### Determination of antioxidant activity as the ability to scavenge DPPH free radicals

The antioxidant activity of the obtained hydrolysates was assessed on the basis of the radical scavenging effect of the stable 1,1-diphenyl-2-picrylhydrazyl [DPPH (Sigma, D21140-0)]—free radical activity according to Yen and Chen (1995), with minor modifications. The tested samples were dissolved in water to a final volume of 1 mL and mixed with 1 mL of ethanol (98 %). The reaction was started by adding 0.5 mL of 0.3 M DPPH in ethanol. The mixtures were left for 30 min at room temperature and the absorbance of the resulting solutions was measured at 517 nm. For calibration, aqueous solutions of known Trolox concentrations ranging from 2 to 20 μg (able to scavenge 500 μL of 0.3 mM DPPH radical solution) were used. Radical scavenging activity of the peptides was expressed as µM trolox_eq_/mg protein.

### FRAP method

The ferric reducing antioxidant power (FRAP) method was used to determine the antioxidative capacity of hydrolysates according to Benzie and Strain (1996). 3 mL of FRAP working solution [300 mM acetate buffer pH 3.6; 10 mM 2,4,6,tripyridyl-s-triazine (TPTZ) (Fluka, 93285) and 20 mM FeCl_3_ × 6 H_2_O (10:1:1 v/v)] was mixed with 1 mL of the sample. After 10 min of reaction, the absorbance was measured at *λ* = 593 nm. An aqueous solution of known Fe(II) concentration was used for calibration (in the range from 100 to 1,000 μg). Results were expressed as μg Fe^2+^/mg protein.

### Determination of Fe(II) ion chelation

Chelation of iron ions by hydrolysates was estimated by the method of Xu et al. (2007) with modifications. A 250 μL sample was mixed with 1,250 μL H_2_O and 110 μL 1 mM FeCl_2_. After 2 min, 1 mL of 500 μM ferrozine (Sigma 160601) aqueous solution was added and the mixture was allowed to react for 10 min. The absorbance of ferrous iron–ferrozine complex was measured spectrophotometrically at *λ* = 562 nm. A known concentration of FeCl_2_ (0–20 μg) was used to generate a standard curve and the ability to chelate iron ions was expressed as μg Fe^2+^/mg protein.

### α-Glucosidase inhibitory activity

The α-glucosidase inhibition assay was performed according to the method of Yu et al. (2011). α-Glucosidase from *Saccharomyces cerevisiae* (Sigma G0660) hydrolyzes the substrate—p-nitrophenyl glucopyranoside (pNPG) (Sigma N1377) and the thus produced p-nitrophenol can be measured spectrophotometrically. 5 µL of the α-glucosidase solution (10 U/mL, in 0.1 M potassium phosphate buffer, pH 6.8) was premixed with 10 µL of the sample solution at different concentrations (in 10 % DMSO) in 620 µL of 0.1 M potassium phosphate buffer (pH 6.8). Following incubation at 37.5 °C for 20 min, 10 µL of p-nitrophenyl glucopyranoside (pNPG 10 mM) as substrate was added to the mixture to start the reaction and was then incubated at 37.5 °C for 30 min, followed by addition of 650 µL of 1 M Na_2_CO_3_ solution to terminate the reaction. The amount of released product (p-nitrophenol) was measured at *λ* = 410 nm. The IC_50_ value was defined as the concentration of inhibitor required to inhibit 50 % of α-glucosidase activity under assay conditions.

### Dipeptidyl peptidase-4 (DPP-4) inhibitory activity

DPP-4 inhibitory activity (IC_50_) was determined using a modified method of Lacroix and Li-Chan (2013). DPP-4 from porcine kidney was purchased from Sigma (D7052). The lyophilized peptide fractions were re-suspended in 0.1 M Tris–HCl buffer, pH 8.0. The test sample (25 μL) was preincubated with an equal volume of the substrate Gly-Pro-p-nitroanilide (1.6 mM) at 37 °C for 10 min. Afterwards, 50 μL of DPP-4 (0.01 U/mL, in 0.1 M Tris–HCl buffer, pH 8.0) was added and the mixture was incubated at 37 °C for 60 min. The reaction was stopped by the addition of 100 μL of 1 M sodium acetate buffer, pH 4.0. The released p-nitroanilide as a hydrolysis product was measured at *λ* = 405 nm.

The IC_50_ value was defined as the concentration of inhibitor required to inhibit 50 % of DPP-4 activity under assay conditions.

#### Ion-exchange chromatography

First, to remove unhydrolyzed protein (mainly phosvitin), the peptic hydrolysate of YP was precipitated [pH adjustment to 4.0 left overnight at 4 °C, then centrifuged (5,500*g*, 10 min, 4 °C)], and the obtained supernatant was used further. Ion-exchange chromatography was performed using a SP Sephadex c-50 resin (Pharmacia Fine Chemicals, 1569) (column size 1.5 × 11.0 cm) with a BioLogic LP (Bio-Rad) system. The operation conditions were as follows: phase A: 0.02 M acetate buffer pH 3.5; phase B: 0.02 M acetate buffer pH 3.5 with 1 M NaCl, gradient: 0.5 % B/min; flow rate: 0.5 mL/min; and fraction volume: 2 mL. The absorbance of eluents was monitored at *λ* = 235 nm.

### Reversed-phase high-performance liquid chromatography

Peptide profiles of hydrolysates were estimated by reversed-phase high-performance liquid chromatography (RP-HPLC) with an Agilent 1100 Series system. Separation was performed using a Zorbax XDB-C 18 Agilent column [4.5 × 250 mm (for fraction 4), 1.8 × 50 mm (for peptic hydrolysate and fraction 4.3)] at *T* = 30 °C. The starting phase (A) was 0.1 % TFA (Fluka) in water and the eluting phase (B) was 0.1 % TFA in acetonitrile (LabScan). The other operation conditions were varied; peptic hydrolysate: flow rate 1 mL/min, gradient: from the second minute of analysis 5 % B/min; fraction 4: flow rate: 1 mL/min, gradient: 5 min 0 % B, 5–45 min 15 % B, 45–50 min 30 % B, fraction 4.3: flow rate: 0.5 mL/min, gradient: 2 min 0 % B, 2–22 min 10 % B, 22–30 min 15 % B. The absorbance of eluents was monitored at *λ* = 235 nm.

RP-HPLC purification and analysis of peptides obtained by the chemical synthesis were carried out using Varian ProStar and Thermo Separation Product liquid chromatographs. Separations were performed on a YMC-Pack ODS-AQ12S05-2546WT column with a Guard Column and a TSKgel ODS-120T 12TG08eh004 column also equipped with a Guard Column, with a linear elution gradient of 0–80 % B in A: A (0.1 % TFA in water) and B (80 % CH 3 CN in water + 0.1 % TFA), at flow rates of 7 mL/min and 1 mL/min for the Varian ProStar and Thermo Separation Product equipment, respectively.

### Determination of peptide molecular weight and sequence and analysis of chemically synthesized peptides

The peptide fraction obtained by RP-HPLC was lyophilized. Then the sample was dissolved in 1 mL 60 % acetonitrile, centrifuged and evaporated. The peptide sample was dissolved again in 60 μL 60 % acetonitrile. 40 μL of this solution was evaporated to dryness in a centrifugal evaporator and was then dissolved in 10 µL of 50 % (v/v) acetonitrile. 0.5 µL of this peptide solution was then mixed with 0.5 µL of alpha-cyano-4-hydroxycinnamic acid solution, pipetted onto a steel plate and evaporated to dryness. MALDI-ToF analysis was performed using UltrafleX-treme apparatus (Bruker Daltonics, Germany) in positive-ion mode. The MS and MS/MS spectra were collected and processed using FlexControl, FlexAnalysis, BioTools as well as ProteinScape software (Bruker Daltonics, Germany), while protein sequences were identified using a MASCOT server (Matrix Science, USA) and the UniProtKB/SwissProt protein sequences database (version 25.10.2012, European Bioinformatics Institute, UK). During searching, the taxonomy of the source organism was restricted to *Gallus gallus,* and to the possible modifications of polypeptide chains to methionine oxidation. No restrictions concerning peptidase specificity during peptide processing were declared.

Analysis of the chemically synthesized peptides was carried out using a HR-ESI-MS (FT-ICR Apex-Qe Ultra 7 T, Bruker Daltonics, Bremen, Germany) equipped with a standard electrospray ion source. The instrument was operated in the positive-ion mode. The instrument was calibrated using a TuneMix™ mixture (Bruker Daltonics, Bremen, Germany). The solution used for the measurements was CH_3_CN/H_2_O/HCOOH (50: 50: 0.1, v/v/v), with a range of values from 100 to 1,800 *m/z*.

### Chemical synthesis of the peptides

Chemical synthesis of the peptides was carried out at the Faculty of Chemistry at the University of Wrocław (Poland). The synthesis of specific peptides was carried out manually in a syringe reactor (BRAUN Inject, Germany). Preloaded Wang resins (0.58–0.79 mM/g) (Iris Biotech GmbH) were used for the synthesis of fully protected peptides. Fmoc-protecting group (9-fluorenylmethyloxycarbonyl) was removed with 25 % piperidine solution (Sigma-Aldrich) in DMF (3 and 17 min) (Carl Roth GmbH + Co. KG). Amino acid coupling reaction was carried out using DMF as a solvent with the use of 3 equivalents TCTU (Iris Biotech GmbH) as the coupling reagent, three equivalents of HOBt [GL Biochem (Shanghai) Ltd.], and six equivalents of DIEA (Iris Biotech GmbH) as additives. The reactions were carried out for 150 min. Peptides were cleaved from the resin simultaneously with side chain deprotection using a mixture of TFA (Iris Biotech GmbH)/TIS (Alfa Aesar)/H_2_O (95: 2.5: 2.5, v/v/v). The reaction was carried out for 120 min. Then, the solution was transferred into cold diethyl ether (Sigma-Aldrich). The crude residue was collected, dissolved in water, lyophilized and purified by reversed-phase high-performance liquid chromatography. All peptides were obtained as their trifluoroacetate salts.

### Statistical analysis

Each type of hydrolysates was prepared in two independent batches. The biological activity measurements of hydrolysates as well as various fractions obtained in the purification process were done in triplicate for each batch. Statistical significance of the differences was determined by the Duncan’s *t* test (*p* < 0.05).

## Results and discussion

### Preparation of biologically active peptides from the egg yolk protein by-product

**Fig. 1:**
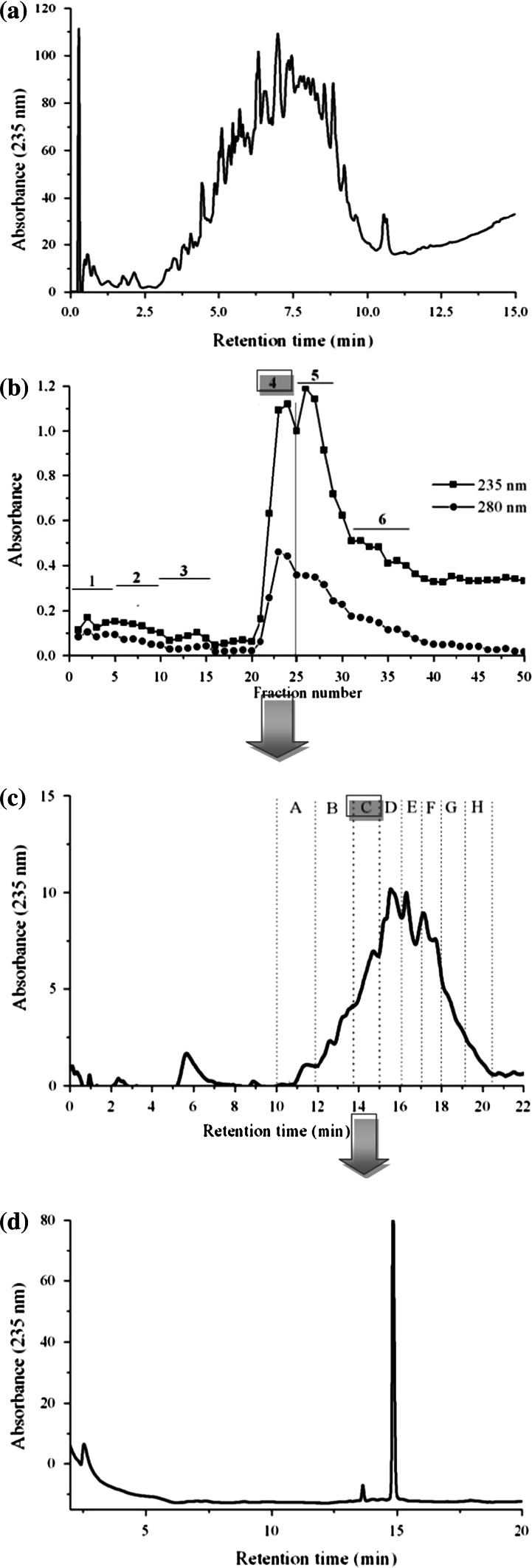
Scheme of peptide isolation from peptic egg yolk protein by-product (YP) hydrolysate. **a** Peptide profile (RP-HPLC) of 2 h hydrolysate; **b** ion-exchange chromatography; **c** RP-HPLC of fraction no 4; **d** re-chromatography (RP-HPLC) of fraction C

Numerous studies have been undertaken to develop novel, natural and safe biologically active peptides from food protein sources. However, only a few researchers have focused on protein by-products as a fairly inexpensive source of biopeptides. One such protein waste is delipidated egg yolk protein (YP), a by-product of phospholipid extraction from egg yolk (Ting et al. 2011; Siepka et al. 2010). In this study, YP was hydrolyzed with porcine pepsin. The hydrolysis was necessary to release the antioxidant and ACE inhibitory peptides from the inactive forms of intact YP by-product. After a 2-h reaction the extent of the protein was estimated by assessing the degree of hydrolysis (DH) and peptide profile analysis (RP-HPLC). The hydrolysis rate was 45.3 %. The RP-HPLC profiles of YP peptic hydrolysate were characterized by numerous peaks varying in their hydrophobicity (Fig. 1a). An increase in antioxidant and ACE inhibitory activity was observed as a result of the hydrolysis. The peptic hydrolysate demonstrated significant increases in DPPH free radical scavenging, ferric reducing power (FRAP) and iron chelating activities, achieving values of 0.5 µM Trolox_eq_/mg, 56.4 µg Fe^2+^/mg, and 405.0 µg Fe^2+^/mg, respectively.

The peptic (2-h) hydrolysate of YP possessed a remarkably higher FRAP (12.7 times) and iron chelating (33.8 times) activity than a 4-h peptic hydrolysate obtained in previous work, which may be attributed to the higher DH (2.37 times) (Zambrowicz et al. 2012). The presented results are in accordance with our earlier study in which hydrolysates obtained from a phosvitin protein preparation treated by trypsin and protease from *A. melleus* exhibited a comparable ferric reducing power with values of 24.0 and 31.3 µg Fe^2+^/mg (Zambrowicz and Trziszka 2010).

Our results confirm the observations of other authors that enzymatic hydrolysates of egg yolk proteins may exert stronger antioxidant activity. Peptide fractions (MW <5 kDa) from protein hydrolysate of lecithin free egg yolk prepared with Alcalase exhibited activities as reflected in TBA and PV that were 30 and 43 % better than that for α-tocopherol (Park et al. 2001). Additionally, Sakanaka and Tachibana (2006) reported that hydrolysates prepared by sequential hydrolysis of egg yolk, conducted with first orientase and then with protease from *Bacillus ssp*., showed strong antioxidant activity. DPPH and hydroxyl radical scavenging activities (at 0.5 % of the hydrolysates) were 74.2 and 91.7 %, respectively.

The peptic hydrolysate of YP also exhibited a significant ACE inhibitory activity (IC_50_ = 623.0 μg/mL). The IC_50_ value of the peptic hydrolysate of YP was not comparable to other values reported for enzymatic hydrolysates of egg yolk proteins. The level of this activity for egg yolk hydrolysates prepared with the use of gastrointestinal (pepsin, pancreatin) and non-gastrointestinal (thermolysin, alkalase) proteases ranged from 133.4 to 210.2 µg/mL (You and Wu 2011). The different levels of antihypertensive activity in our study might have resulted from the use of YP by-product instead of whole egg yolk in its native form.

Yoshi et al. (2001) obtained peptides of 1 kDa or less with ACE inhibitory activity by the hydrolysis of egg yolk with a crude enzyme. These peptides act by lowering systolic blood pressure in rats, in a dose-dependent manner, and the study implied that peptides extracted from chicken egg yolk could potentially suppress the development of hypertension in SHR, and this effect might be induced by the inhibition of ACE activity (Yoshi et al. 2001).

On the other hand, our own results showed that peptic YP hydrolysate possessed significantly higher ACE inhibitory activity than hydrolysates obtained from egg white proteins. Fujita et al. (2000) demonstrated that hydrolysates obtained from ovalbumin using pepsin and thermolysin exhibited ACE inhibitory activity with IC_50_ values of 45 and 83 mg/mL, respectively.

### Isolation and purification of bioactive peptide

The peptic hydrolysate of YP was precipitated (pH 4.0) and centrifuged to remove unhydrolyzed protein (mainly phosvitin). The antioxidant and ACE inhibitory activities of the obtained supernatant were significantly higher than the 2-h hydrolysate (Table 1). Then, to find peptides exhibiting a potential biological effect, the obtained supernatant was subjected to ion-exchange chromatography by LC on a SP-Sephadex c-50 resin (Fig. 1b). The procedure allowed for the isolation of six fractions (1–6) which were collected, concentrated by lyophilization and then evaluated for antioxidant and ACE inhibitory activities. Two peptide fractions: 1 (IC_50_ = 249.0 µg/mL) and 4 (IC_50_ = 283.1 µg/mL) with ACE inhibitory activity were obtained (Table 1). Additionally, fraction no. 4 exerted the strongest antioxidant activity among all the obtained fractions, with DPPH free radical scavenging, ferric reducing power (FRAP) and iron chelating activity reaching: 2.9 µM Trolox_eq_/mg, 110.6 µg Fe^2+^/mg, and 250.1 µg Fe^2+^/mg, respectively. This fraction was further purified by RP-HPLC on a Zorbax C18 column and the eluate was divided into eight major fractions (A–H) (Fig. 1c). The analysis of all collected fractions showed a wide diversity in the levels of antioxidant and ACE inhibitory activity. The strongest ACE inhibitory activity (IC_50_ = 35.0 µg/mL) was estimated for fraction C (Table 1). Furthermore, fraction D was also a strong inhibitor and two times higher than that necessary to deactivate 50 % of the enzyme with fraction C. Similar to the previous step of the isolation procedure, only one fraction, C, was characterized by a strong antioxidant activity of; DPPH radical scavenger (3.0 µM Trolox_eq_/mg), ferric reducer (112.1 µg Fe^2+^/mg) and iron chelator (106.5 µg Fe^2+^/mg).

**Table 1:** Biological activities of the peptide fractions separated by precipitation, ion-exchange and reverse-phase chromatography of 2-h egg yolk protein by-product (YP) hydrolysate treated by pepsin

Isolation step	Sample	ACE IC_50_ (µg/mL)	DPPH (µM Trolox_eq_/mg)	FRAP (µg Fe^2+^/m)	CHEL (µg Fe^2+^/mg)
Hydrolysis	Peptic hydrolysate (2 h) (DH 45.3 %)	623.0a	0.5h,i	56.4f	405.0b
Precipitation (pH 4.0; centrifugation)	Supernatant	361.0d	1.6d,e	78.1d	458.6a
Ion-exchange chromatography	1	249.1e	0.54hi	70.5e	13.2h
	2	291.5d	0.55hi	23.3i	94.6f
	3	NA	1.5d	15.1j	109.3f
	4	283.0d	2.9b	110.6c	250.1c
	5	475.8b	1.4e	NA	128.2e
	6	NA	0.67gh	NA	38.3g
RP-HPLC	A	NA	NA	NA	NA
	B	152.3f	1.9c	112.1c	NA
	**C (4.C)**	35.0h	3.0ab	184.2b	406.5b
	D	72.7g	0.8g	76.3de	175.6d
	E	245.9e	0.8g	54.7f	NA
	F	423.0c	1.0f	40.0g	NA
	G	NA	NA	31.2h	NA
	H	NA	0.4i	25.6hi	27.3gh
	4.C.1	29.0h	3.1a	190.8a	402.5b

All data were expressed as mean values (*n* = 3)

Values sharing the same letter are not significantly different at *p* < 0.05

Mean values with different letters in the same column are significantly different at *p* ≤ 0.05

*NA* no activity

Interestingly, our work indicated that peptide fractions from YP hydrolysate possessed strong combined DPPH radical scavenging and ACE inhibitory activities. This was in accordance with a study conducted by Lu et al. (2010), in which enzymatic hydrolysates with high ACE inhibitory activity usually also possessed high levels of DPPH radical scavenging activity. This was also observed by Davalõs et al. (2004) who described the ovalbumin-derived peptide Tyr–Ala–Glu–Glu–Arg–Tyr–Pro–Ile–Leu. It had a strong ACE inhibitory activity, also possessing high radical scavenging activity, and delayed low-density lipoprotein lipid oxidation induced by Cu^2+^ ions.

The chromatogram obtained during further chromatography (RP-HPLC) of fraction C indicated the presence of one main peptide peak (Fig. 1d).

Purification of the YP preparation resulted in a significant increase in ACE inhibitory and DPPH radical scavenging activity compared to the original hydrolysate, indicating the effectiveness of the isolation procedure. At the same time, the proposed procedure had no significant impact on the level of iron chelating or ferric reducing activity.

### Amino acid sequence and characteristics of purified peptides

**Fig. 2:**
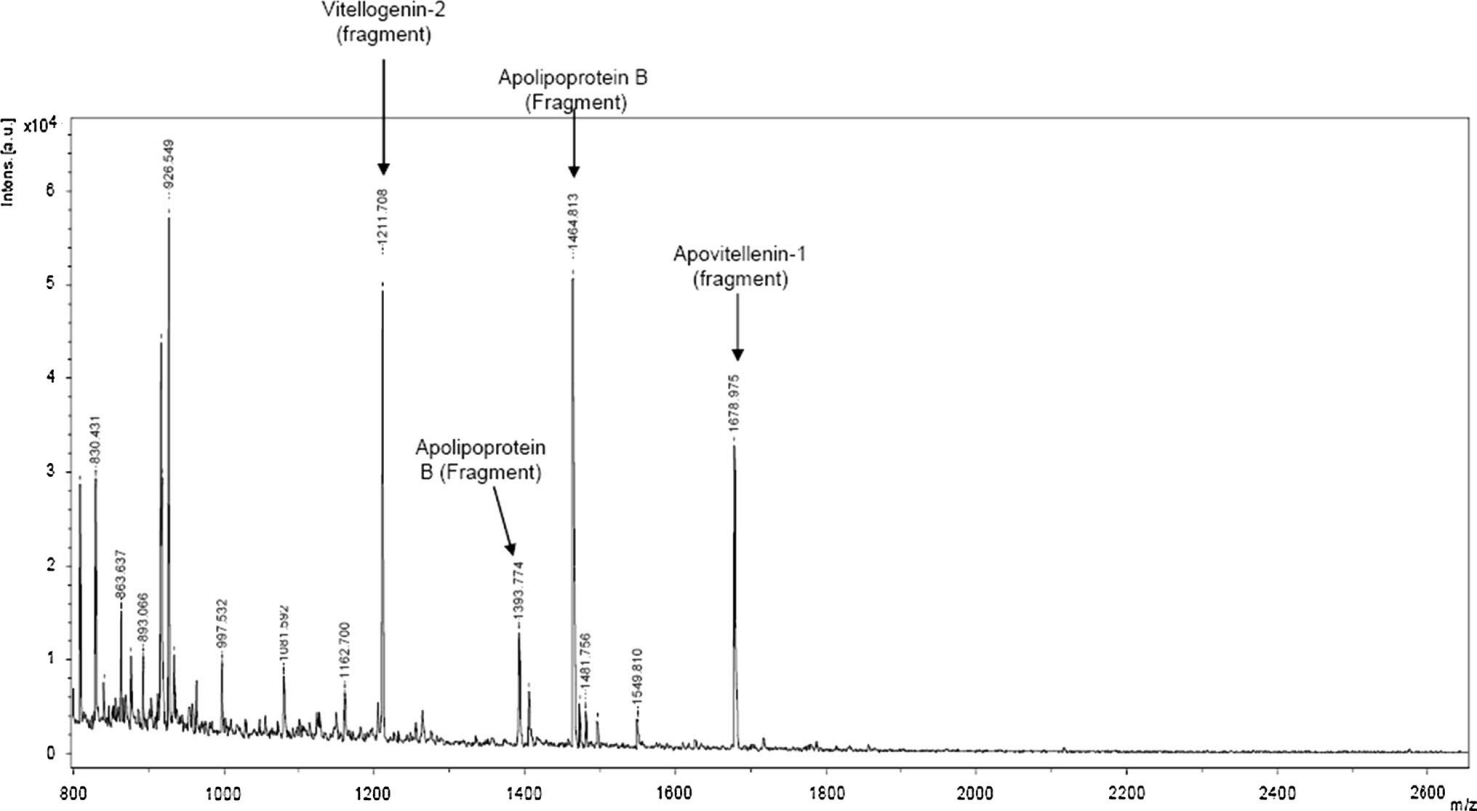
Determination of peptide molecular weight and sequence. MS-Maldi-ToF spectrum of fraction C isolated from 2-h egg yolk protein by-product (YP) hydrolysate

**Fig. 3:**
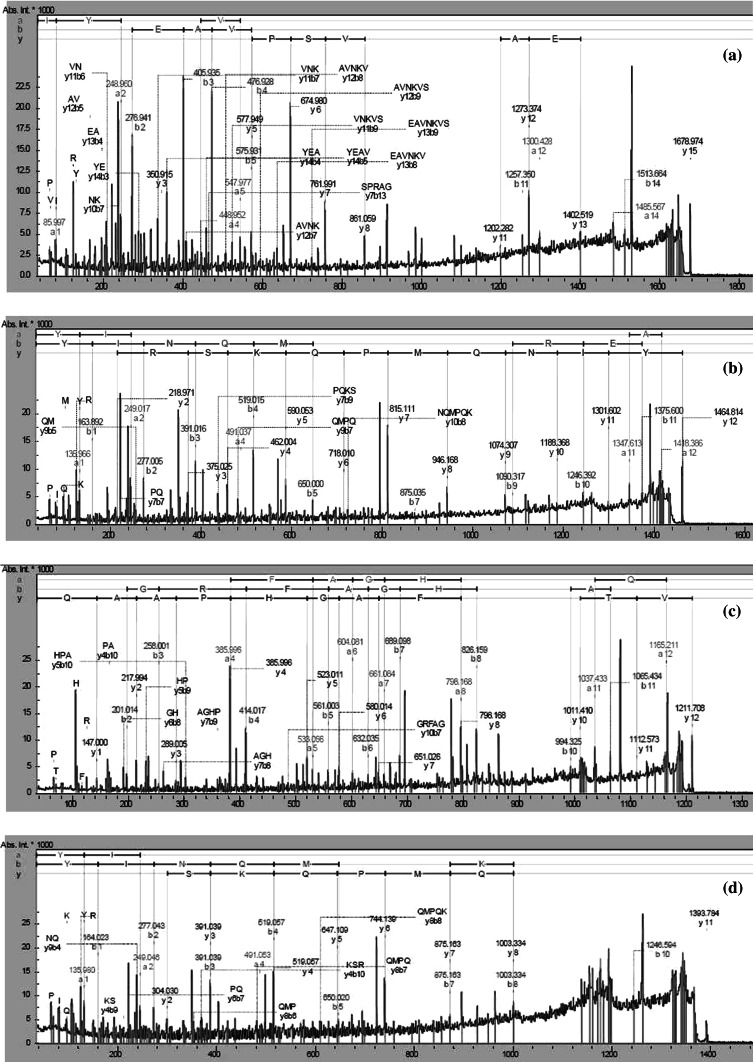
Mascot search results analysis of MS spectrum of peptide fraction C. **a** Apovitellenin-1 peptide: IYEAVNKVSPRAGQF, **b** apolipoprotein B peptide: YINQMPQKSREA; **c** Vitellogenin-2 peptide: VTGRFAGHPAAQ; **d** apolipoprotein B peptide: YINQMPQKSRE, identified in fraction C

Fraction C with the highest antioxidant and ACE inhibitory activity was subjected to MALDI-ToF analysis to identify its amino acid sequence. The MS spectrum presented in Fig. 2 revealed four major signals at *m/z* = 926.549, 1,211.708, 1,464.813 and 1,678.975, respectively, accompanied by a large group of low-intensity signals (among them *m/z* = 1,393.774) below a 1,600 *m/z* value. Unfortunately, the peaks of the substances corresponding to the minor signals were too low to perform MS/MS analysis; therefore, we focused our attention on the major signals at *m/z* = 926.549, 1,211.708, 1,464.813 and 1,678.975. Mascot search result analysis revealed that the signals at *m/z* = 1,393.774 [M+H+]^+^ and *m/z* = 1,464.813 [M+H+]^+^ corresponded to the single-protonated fragments of Apolipoprotein B (YINQMPQKSRE and YINQMPQKSREA, respectively), and the signals at *m/z* = 1,211.708 [M+H+]^+^ and *m/z* = 1,678.975 [M+H+]^+^ corresponded to single-protonated fragments of Vitellogenin-2 (VTGRFAGHPAAQ) and Apovitellenin-1 (YIEAVNKVSPRAGQF), respectively (Fig. 3).

ACE inhibitory peptides usually contain 3–13 amino acid residues (Li et al. 2004). Thus, the molecular weights of most YP-derived peptides were in good agreement with the size of the reported ACE inhibitory peptide.

To confirm antioxidant and ACE inhibitory activity, the YP-derived peptides were synthetized and tested (Table 2). Furthermore, antidiabetic activity, as reflected in α-glucosidase and DPP-IV inhibitory properties, was determined. The resulting peptides were characterized by a high purity (determined by RP-HPLC) of 91–99 % (Table 2). Almost all of them exerted triple activities: antioxidant, ACE-inhibitory and antidiabetic. Individual synthetic peptides showed lower antioxidant activity than those pooled in the mixture (fraction C). In contrast, IC_50_ values of the synthesized peptides were significantly higher than those for fraction C (Tables 1, 3). Peptide YINQMPQKSRE revealed the strongest antioxidant activity. The DPPH free radical scavenging and iron chelating activities reached 2.3 µM Trolox_eq_/mg and 37.4 µg Fe^2+^/mg, respectively. However, peptide YINQMPQKSREA differed with the presence of alanine at the C-terminal sequence, and was characterized by the highest ferric reducing activity (76.0 µg Fe^2+^/mg). Antioxidative peptide YINQMPQKSRE also demonstrated very strong ACE (IC_50_ = 10.1 µg/mL) and DPP-IV (IC_50_ = 222.8 µg/mL) inhibitory activity. Additionally, the level of DPP-IV inhibitory activity for that peptide was the highest among all the tested peptides. The strongest ACE inhibitory activity with IC_50_ = 9.4 µg/mL was yielded by the sequence YIEAVNKVSPRAGQF. This peptide exerted at the same time relatively strong antioxidant activity. The DPPH free radical scavenging, ferric reducing and iron chelating activities reached 2.2 µM Trolox_eq_/mg, 61.0 and 25.0 µg Fe^2+^/mg, respectively. However, peptide YIEAVNKVSPRAGQF, which was characterized as the most effective ACE inhibitor, had no impact on the activity of α-glucosidase or DPP-IV.

**Table 2:** Synthetic peptide data

Sequence	MS analysis		RP-HPLC analysis	
	*m/z*	*m/z* calculated	Retention time (min)	Purity (%)
YINQMPQKSREA	1,464.7343	1,464.7269 [M+H]^+^	13.948	96.0
	732.8655	732.8674 [M+2H]^2+^		
	488.9141	488.9142 [M+3H]^3+^		
VTGRFAGHPAAQ	1,211.6318	1,211.6285 [M+H]^+^	12.948	97.0
	606.3164	606.3182 [M+2H]^2+^		
	404.5485	404.5481 [M+3H]^3+^		
YINQMPQKSRE	1,393.6923	1,393.6898 [M+H]^+^	13.243	91.0
	697.3487	697.3488 [M + 2H]^2+^		
	465.2355	465.2352 [M + 3H]^3+^		
IYEAVNKVSPRAGQF	1,678.8604	1,678.8971 [M+H]^+^	19.557	99.0
	839.9484	839.9498 [M+2H]^2+^		
	560.3026	560.3024 [M+3H]^3+^

**Table 3:** Biological activity of synthetic peptides

Peptide	Enzyme inhibitory activity (IC_50_)			Antioxidant activity		
	ACE (µg/mL)	α-Glucosidase (µg/mL)	DPP-4 (µg/mL)	DPPH (µM Trolox_eq_/mg)	FRAP (µg Fe^2+^/mg)	CHEL. (µg Fe^2+^/mg)
Y**I**NQ**MP**QKS**R**E	10.1a	1,694.3a	222.8a	2.3c	59.0b	37.0a
Y**I**E**AV**NK**V**S**PRAG**Q**F**	9.4a	NA	NA	2.2c	61.0b	25.2b
Y**I**NQ**MP**QKS**R**E**A**	12.6b	454.6b	355.8b	1.8b	76.0a	8.5c
**V**T**GRFAGHPAA**Q	27.3c	365.4c	1,402.2c	1.5a	58.0b	NA

All data were expressed as mean values (*n* = 3)

Values sharing the same letter are not significantly different at *p* < 0.05

Mean values with different letters in the same column are significantly different at p ≤ 0.05

The bolded letters at the peptide sequence represent hydrophobic and positively charged residues

*NA* no activity

The highest level of α-glucosidase inhibitory activity (IC_50_ = 365.4 µg/mL) was exhibited by peptide VTGRFAGHPAAQ.

Representative examples of multifunctional food-derived peptides are fragments obtained during gastrointestinal digestion of lysozyme (Rao et al. 2012). These peptides exert strong competitive ACE inhibitory and remarkable antioxidant activity. Some other multifunctional peptides can be released from milk proteins. Peptides from sequence 60–70 of β-casein show immunostimulatory, opioid and ACE inhibitory activities (Sharma et al. 2011).

According to Rao and others (2012), proteins containing a high proportion of hydrophobic and positively charged residues have the potential to generate multifunctional peptides. The percentage content of hydrophobic and positively charged residues in YP-derived peptides was in the range from 36.1 to 75.0 % (Table 3).

Among several of the amino acids, tyrosine is generally accepted as an antioxidant in spite of a pro-oxidative effect. Commonly, among the analyzed peptides herein with the strongest antioxidant activity is the presence of tyrosine at the N-terminus or at the second position of the N-terminal sequence. In contrast, His-containing peptide VTGRFAGHPAAQ exerted the lowest antioxidant activity. His-containing peptide activity may be attributed to a hydrogen-donating ability, peroxyl radical trapping and/or the metal ion-chelating ability of the imidazole group. It may be speculated that the His residue in the peptide shows no reactivity against oxidation without the C-terminal segment. For example, the deletion of His residue located at the C-terminal in the amino acid sequence from synthetic analogs of peptides isolated from soybean protein decreased their antioxidant activity (Chen et al. 1996).

Substances with ACE inhibitory potency are mainly zinc chelators (or either a thiol, phosphate, carboxylate oxygen), hydrogen-bond and carboxyl-terminal group donors. Therefore, the amino acid sequence strongly affects the potential ACE inhibition. According to FitzGerald, Murray and Walsh (2004), a glutamic acid residue at the C-terminal positions additionally contributes to the ACE inhibitory potency of the peptides. This is related to the ability of Glu to chelate zinc ions, a component of the active site of the angiotensin-converting enzyme. This may explain the high activity of peptide YINQMPQKSRE (IC_50_ = 10.1 µg/mL).

According to reports from other authors, particular peptides with hydrophobic amino acids (aromatic or branched side chains) in the sequence take part in the inhibition of ACE (Kumar et al. 2010). Moreover, as the activity of ACE is to cleave the C-terminal dipeptide of oligopeptide substrates, the inhibitory potential of peptides is strongly influenced by their C-terminal tripeptide sequences (Ni et al. 2012). The most potent ACE inhibitors contain hydrophobic amino acid residues at their three C-terminal positions (Rohrbach et al. 1981). In our study, peptide IYEAVNKVSPRAGQF contained two valine residues in the sequence and phenylalanine residues at the C-terminal tripeptide sequence, and all the isolated peptides contained one proline in their sequence.

Food components with α-glucosidase and DPP-IV inhibitory activities may represent nutraceutical effect on diabetes. Alkaline protease hydrolysates from sardine muscle (IC_50_ = 48.7 mg/mL), chicken essence (IC_50_ = 471.4 mg/mL) or yogurt (IC_50_ = 519.8 mg/mL) showed potent α-glucosidase inhibitory activity (Matsui et al. 1996). Recently, Yu et al. (2012) described eight novel antidiabetic peptides from ovalbumin, amongst them the most potent peptide KLPGF had α-glucosidase inhibitory activity of IC_50_ = 59.5 µmol/L. Our results indicate that egg yolk proteins also may be a worthwhile source of peptides with α-glucosidase and DPP-IV inhibitory activity.

To date, no clear dependencies have been found between the structure and activity of peptides with potential α-glucosidase and DPP-IV inhibitory activities due to the limited knowledge of the chemical structures of those peptides.

## Conclusion

The purification and characterization of egg yolk protein by-product, from the ethanol extraction of phospholipids (YP) derived peptides, by precipitation, ion-exchange, RP-HPLC and MALDI-ToF mass spectrometry has lead to the detection of peptide sequences: YINQMPQKSRE, YIEAVNKVSPRAGQF, YINQMPQKSREA and VTGRFAGHPAAQ. To validate the biological activity of the YP-derived peptides, they were chemically synthesized. In the course of our studies on antioxidant, ACE inhibitory and antidiabetic activities (with α-glucosidase and DPP-4 inhibitory activity), we have successfully revealed that peptide YIEAVNKVSPRAGQF showed mild inhibitory activity against ACE (IC_50_ = 9.4 µg/mL). Peptide YINQMPQKSRE showed the strongest antioxidant and DPP-IV inhibitory activities The DPPH free radical scavenging, iron chelating and DPP-IV inhibitory activity reached: 2.3 µM Trolox_eq_/mg and 37.4 µg Fe^2+^/mg and IC_50_ = 222.8 µg/mL, respectively. Peptide VTGRFAGHPAAQ showed significant antidiabetic activity against α-glucosidase (IC_50_ = 365.4 µg/mL). The current study suggests that peptides from YP have the potential as suitable candidates for further investigations as natural and multifunctional substances to control diet-related diseases.
